# Proactive Management of Gestational Hypertension in a Patient With Uterine Fibroids: A Case Report

**DOI:** 10.1002/ccr3.72536

**Published:** 2026-04-12

**Authors:** Fazeela Bibi, Mala Jitendra Shahani, Muhammad Ibrahim, Khalil Elabdi, Bilal Aslam, Barkah Ali, Usama Khan, Romaisa Naveed Akhtar, Nazia Moazzam, Mantasha Gauhar, Said Hamid Sadat

**Affiliations:** ^1^ Jinnah Medical and Dental College Karachi Pakistan; ^2^ Dow Medical College Karachi Pakistan; ^3^ Bannu Medical College Bannu Pakistan; ^4^ Faculty of Medicine and Pharmacy of Rabat, Mohammed V University Rabat Morocco; ^5^ University of Lahore Lahore Pakistan; ^6^ Nowshera Medical College Nowshera Pakistan; ^7^ Liaquat National Medical College Karachi Pakistan; ^8^ Sir Syed College of Medical Sciences for Girls Karachi Pakistan; ^9^ Jinnah Sindh Medical University Karachi Pakistan; ^10^ Kabul University of Medical Science Abu Ali Ibn Sina Kabul Afghanistan

**Keywords:** anticipatory care, case report, gestational hypertension, high‐risk pregnancy, home blood pressure monitoring, leiomyoma, low‐dose aspirin, methyldopa, proactive management, uterine fibroids

## Abstract

The coexistence of uterine fibroids and gestational hypertension (GH) presents a high‐risk obstetric scenario associated with adverse maternal‐fetal outcomes. We present the case of a 27‐year‐old primigravida diagnosed with a 22 × 28 mm anterior uterine fibroid at 8 weeks' gestation, which enlarged to 52 × 48 mm by 29 weeks. At 21 weeks' gestation, she developed new‐onset GH (blood pressure 150/98 mmHg) without proteinuria. Her management included prophylactic low‐dose aspirin (75 mg daily), methyldopa (250 mg twice daily), and structured home blood pressure monitoring. When her blood pressure rose to 155/100 mmHg at 34 weeks, the methyldopa dosage was increased to three times daily, which successfully restored control. Following induction of labor at 40 weeks, the patient had an uncomplicated vaginal delivery of a healthy 3.3 kg neonate with an APGAR score of 9. The postpartum course was unremarkable, and the fibroid regressed significantly. This case illustrated that a proactive, guideline‐based strategy uniting early prophylaxis, adaptive pharmacotherapy guided by home monitoring, and anticipatory care can successfully navigate the compounded risks of this clinical dyad, leading to a favorable outcome.

## Key Clinical Message

An integrated strategy of low‐dose aspirin prophylaxis, adaptive antihypertensive therapy guided by home monitoring, and anticipatory care can achieve a favorable outcome in high‐risk pregnancies complicated by uterine fibroids and gestational hypertension.

## 1 Introduction

Uterine leiomyomas (fibroids) are the most prevalent benign uterine tumors in reproductive‐aged women, with an estimated incidence in pregnancy as high as 16.7%. This figure is expected to rise with global trends toward delayed childbearing [1, 2]. Concurrently, gestational hypertension (GH)—defined as new‐onset blood pressure elevation (≥ 140/90 mmHg) after 20 weeks of gestation without proteinuria—is a significant obstetric complication affecting 5%–10% of pregnancies worldwide and is independently associated with adverse maternal and perinatal outcomes [3].

A substantial body of evidence demonstrates that the presence of uterine fibroids is a significant independent risk factor for the development of hypertensive disorders of pregnancy (HDP), including GH and preeclampsia [4]. This high‐risk clinical dyad compounds the risks of adverse outcomes, which include spontaneous abortion, preterm labor, fetal growth restriction, and postpartum hemorrhage [2]. Consequently, the effective management of these concurrent conditions requires a comprehensive, proactive, and multidisciplinary approach.

While guidelines exist for managing GH and monitoring fibroids separately, there is significant educational value in case‐based reports that demonstrate how to successfully integrate these protocols in a real‐world clinical scenario. This report presents the case of a primigravida with a large uterine fibroid who developed GH. It serves as a practical, educational example of how a synthesized management strategy—uniting early prophylaxis, adaptive pharmacotherapy, and patient‐centered monitoring—can successfully mitigate these compounded risks to achieve a favorable maternal‐fetal outcome.

## 2 Case Description

A 27‐year‐old primigravida (G1P0) with a notable family history of preeclampsia but no other comorbidities presented for her initial antenatal consultation at 8 weeks' gestation. Her booking blood pressure was 125/80 mmHg and her Body Mass Index was 25 kg/m^2^. A routine prenatal ultrasound confirmed a viable intrauterine pregnancy and incidentally identified a 22 × 28 mm uterine fibroid on the anterior uterine wall.

A 20‐week anomaly scan confirmed appropriate fetal development and demonstrated significant interval growth of the fibroid, which now measured 46 × 40 mm. At a routine follow‐up at 29 weeks' gestation, ultrasound imaging revealed further fibroid enlargement to 52 × 48 mm.

## 3 Diagnostic Investigations and Differentials

At a scheduled visit at 21 weeks' gestation, the patient's blood pressure was recorded at 150/98 mmHg. She was asymptomatic. To confirm the diagnosis of new‐onset hypertension, she was observed in the clinic for six hours. The diagnosis was confirmed with four additional readings over the six‐hour period, all of which remained consistently above 140/90 mmHg.

A comprehensive laboratory workup was performed, including a complete blood count, liver function tests, serum creatinine, and urinalysis for proteinuria; all results were within normal limits. Based on new‐onset blood pressure elevation (≥ 140/90 mmHg) after 20 weeks' gestation without proteinuria or other signs of end‐organ damage, a definitive diagnosis of gestational hypertension (GH) was established. The primary differential diagnosis of preeclampsia was clinically excluded.

At 34 weeks' gestation, the patient's blood pressure increased to 155/100 mmHg during a routine visit. A repeat laboratory evaluation at that time again showed no evidence of proteinuria or other severe features, reinforcing the diagnosis of GH.

## 4 Treatment, Outcomes and Follow–Up

Following the diagnosis of GH at 21 weeks' gestation, a multi‐faceted management plan was initiated. This included prophylactic low‐dose aspirin (75 mg daily) and first‐line antihypertensive therapy with oral methyldopa (250 mg twice daily). The patient was also equipped for and educated on a structured home blood pressure monitoring (HBPM) regimen. The patient's home blood pressure device was validated against a calibrated clinic sphygmomanometer prior to initiating monitoring.

Her blood pressure remained well‐controlled within the target range until the 34‐week visit. In response to the recorded pressure of 155/100 mmHg, the methyldopa frequency was promptly increased to 250 mg three times daily. This adaptive therapeutic adjustment successfully restored her blood pressure to a target range of 125–130/80 mmHg, which was maintained for the remainder of the pregnancy.

At 40 weeks' gestation, labor was induced for obstetric indications. The induction was performed using a prostaglandin E2 cervical insert to promote cervical ripening, followed by an oxytocin infusion. The patient had an uneventful vaginal delivery of a healthy male infant weighing 3.3 kg, with an APGAR score of 9 at five minutes. The postpartum course was uncomplicated, with no postpartum hemorrhage. A six‐week postpartum pelvic ultrasound confirmed significant fibroid regression to 32 × 30 mm.

## 5 Discussion

This case report provides a practical illustration of how a high‐risk pregnancy involving both uterine fibroids and gestational hypertension can be managed to a successful outcome. The success of the management strategy rested on the synergistic integration of three core evidence‐based principles: early prophylaxis, adaptive pharmacotherapy, and patient‐centered monitoring [3].

The presence of uterine fibroids is recognized as a significant risk factor for hypertensive disorders of pregnancy (HDP), with some studies reporting adjusted hazard ratios as high as 2.95 [4]. The decision to initiate low‐dose aspirin prophylaxis at the time of GH diagnosis was therefore critical, aligning with recommendations from leading bodies like the American College of Obstetricians and Gynecologists (ACOG) for patients with multiple risk factors for preeclampsia [5]. This single intervention likely played a key role in preventing progression to more severe disease. The management of the patient's hypertension was not static but adaptive—a crucial principle in managing volatile hypertensive disorders. Methyldopa, a first‐line agent with a well‐established safety profile in pregnancy, was initiated promptly [6]. The transient hypertensive episode at 34 weeks was met with a decisive and timely dose titration, a response enabled by the availability of reliable data from structured home blood pressure monitoring (HBPM). Evidence from trials such as CHIPS supports that tight, responsive blood pressure control is vital for preventing maternal end‐organ damage while ensuring adequate fetal perfusion [7]. Furthermore, HBPM is increasingly recognized not only for improving maternal blood pressure control but also for enhancing patient engagement, allowing for the data‐driven adjustments that were pivotal in this case [8]. While the precise pathophysiology linking fibroids and HDP is multifactorial and remains under investigation, proposed mechanisms include compromised uteroplacental perfusion due to myomas, leading to localized ischemia [9].

This case does not establish causality but provides a clear clinical example where a known anatomical risk factor (fibroid) was followed by a functional complication (GH). The management focused on mitigating the functional risk, proving to be an effective strategy. The primary limitation of this report is that it describes a single case, and its outcomes cannot be generalized. However, its educational value lies in demonstrating the real‐world application of established guidelines. It reinforces that uterine fibroids should be considered a sentinel risk factor for HDP, prompting intensified surveillance and a low threshold for initiating prophylactic and therapeutic measures throughout gestation.

## 6 Conclusion

This case demonstrates that the compounded risks of uterine fibroids and gestational hypertension can be successfully navigated with a proactive, multi‐faceted management plan. The integration of early aspirin prophylaxis, adaptive pharmacotherapy guided by validated home monitoring, and anticipatory care provides a practical, evidence‐based roadmap to convert a high‐risk clinical profile into a successful maternal‐fetal outcome.

## Author Contributions

**Fazeela Bibi:** conceptualization, investigation, methodology, project administration, resources, supervision. **Mala Jitendra Shahani:** conceptualization, data curation, validation. **Muhammad Ibrahim:** conceptualization, formal analysis, writing – original draft. **Khalil Elabdi:** conceptualization, data curation, formal analysis, investigation, methodology, project administration, resources, supervision, validation, visualization, writing – original draft, writing – review and editing. **Bilal Aslam:** conceptualization, formal analysis, investigation, methodology. **Barkah Ali:** conceptualization, supervision, validation. **Usama Khan:** investigation, methodology, project administration. **Romaisa Naveed Akhtar:** conceptualization, formal analysis, methodology. **Nazia Moazzam:** data curation, investigation, methodology. **Mantasha Gauhar:** data curation, investigation, methodology, resources, writing – original draft. **Said Hamid Sadat:** validation.

## Funding

The authors have nothing to report.

## Consent

Written informed consent was obtained from the patient to publish this report in accordance with the journal's patient consent policy.

## Conflicts of Interest

The authors declare no conflicts of interest.

## Data Availability

Data is available upon reasonable request from the authors.
